# The validity of the non-exercise activity thermogenesis questionnaire evaluated by objectively measured daily physical activity by the triaxial accelerometer

**DOI:** 10.1186/2052-1847-6-27

**Published:** 2014-07-05

**Authors:** Hidetaka Hamasaki, Hidekatsu Yanai, Masafumi Kakei, Mitsuhiko Noda, Osamu Ezaki

**Affiliations:** 1 Department of Internal Medicine, National Center for Global Health and Medicine Kohnodai Hospital, Chiba, Japan; 2 Division of Complementary Medicine, First Department of General Medicine, Saitama Medical Center, Jichi Medical University School of Medicine, Saitama, Japan; 3 Department of Diabetes Research, Diabetes Research Center, National Center for Global Health and Medicine, Tokyo, Japan; 4 Department of Human Health and Design, Faculty of Human Life and Environmental Sciences, Showa Women’s University, Tokyo, Japan

**Keywords:** Physical activity, Non exercise activity thermogenesis, Accelerometer, Type 2 diabetes

## Abstract

**Background:**

Physical inactivity is a major cardiovascular risk factor. Recently, we showed that non-exercise activity thermogenesis (NEAT) assessed by the self-reported questionnaire is favorably associated with metabolic risks in patients with type 2 diabetes. The purpose of the present study was to examine the validity of the questionnaire by comparing with objectively measured daily physical activity (PA) by using the triaxial accelerometer.

**Methods:**

Daily physical activity level (PAL) of 51 participants (24 men and 27 women) with type 2 diabetes was measured by the triaxial accelerometer. At the same time, we evaluated their NEAT score using our original questionnaire modified from a compendium of physical activities.

**Results:**

The NEAT score was significantly and positively correlated with PAL measured by the triaxial accelerometer (r = 0.604, P < 0.001). PAL was also significantly and positively correlated with both the locomotive NEAT score and the non-locomotive NEAT score (r = 0.444, P = 0.001 and r = 0.526, P < 0.001, respectively).

**Conclusions:**

The NEAT score measured by the self-reported questionnaire was highly correlated with PAL measured by the triaxial accelerometer. Our original NEAT questionnaire may be useful for evaluation of daily PAL in clinical practices.

## Background

A sedentary lifestyle is a major cardiovascular risk factor [1] and daily physical activity (PA) is associated with a reduction in all-cause mortality and cardiovascular diseases (CVD) risk in patients with diabetes [2]. Only about 30% of Japanese adults fulfill the recommended physical activity level (PAL) [3], therefore, it is important to understand whether daily PA including going to work, washing clothes and cleaning floors, defined as non-exercise activity thermogenesis (NEAT) [4] can improve CVD risk factors, or not [5,6].

We have previously demonstrated that NEAT score determined by our original questionnaire is favorably associated with insulin sensitivity, waist circumference, high-density lipoprotein cholesterol (HDL-C), blood pressure in patients with type 2 diabetes [7]. However, our study had the limitation that the NEAT score calculated with the self-reported questionnaire is subjective data and may not always represent the true NEAT. Here we investigated the correlation between the NEAT score and PAL measured by the triaxial accelerometer under free living conditions in patients with type 2 diabetes and confirmed the validity of our NEAT questionnaire.

## Methods

### Study participants

This study was approved by the Ethical Committee of the National Center for Global Health and Medicine (reference number NCGM-G-001212-00). All participants provided written informed consent. The participants studied were 51 patients with type 2 diabetes without physical disability aged between 27 and 79 years old. Participants who engaged in active sports-like exercise and resistance training were excluded. Characteristics of the participants studied are shown in Table 1.

**Table 1:** Participants characteristics

**Demographics**	
N	51
Sex (men/women)	24 / 27
Age (years)	58.5 ± 12.3
Height (cm)	160.7 ± 8.7
Weight (kg)	69.0 ± 14.4
BMI (kg/cm^2^)	26.7 ± 5.2
**Physiological parameters**	
PAL	1.62 ± 0.19
BMR (kcal/day)	1344.1 ± 268.5
**Biochemical parameters**	
Fasting plasma glucose (mg/dl)	123.5 ± 25.9
HbA1c (%)	6.7 ± 1.0
**NEAT score**	
Locomotive activities	19.5 ± 4.0
Non-locomotive activities	43.7 ± 9.7
Total	63.2 ± 11.6

Data are means ± SD. BMI, body mass index; PAL, physical activity level; BMR, basal metabolic rate; NEAT, non-exercise activity thermogenesis.

### Daily physical activity measurement by the triaxial accelerometer

Daily PA was measured using the triaxial accelerometer (Active Style Pro HJA-350IT, Omron Co., Ltd, Kyoto, Japan), 74 × 46 × 34 mm and 60 g including batteries. Participants studied wore the accelerometer on the left side of the waist. Anteroposterior, mediolateral and vertical acceleration measurements were obtained during each physical activity at a rate of 32 Hz to 12-bit accuracy. Each of three signals from the triaxial accelerometer was passed through a high-pass filter with a cut-off frequency of 0.7 Hz to remove the gravitational acceleration component. The ratios of unfiltered to filtered total acceleration (TAU/TAF) and filtered vertical and horizontal acceleration (VAF/HAF) were calculated to determine the cut-off value for the classification of locomotive activities and non-locomotive activities including such as household and occupational activities, which resulted in almost 100% accurate demarcation for daily eleven different activities [8]. Furthermore, metabolic equivalent values (METs) determined by the triaxial accelerometer have been reported to be closely correlated with METs calculated by using energy expenditure (EE) measured by the indirect calorimetry [8,9]. Participants studied wore the accelerometer on the left side of the waist for consecutive 7 days, and physical activities were recorded. Participants were requested to wear the accelerometer except under special circumstances such as sleeping, bathing and during aquatic activities. Activity data were stored on a minute-by-minute basis and were downloaded to a personal computer before analysis. We excluded days in which participants did not wear the accelerometer for more than 8 hours from the data for analysis.

Basal metabolic rate (BMR) was estimated from multiple regression equation including age, sex, height and ideal body weight (IBW) as variables, the equation as follows: BMR (kcal/day) = [(0.1283 + 0.0481 × IBW (kg) + 0.0234 × height (cm) - 0.0138 × age (year) - 0.5473 × sex coefficient (man: 1, woman:2)) × 293] [10]. Total energy expenditure (TEE) was calculated by manufactured regression equation using METs assessed by the triaxial accelerometer [9]. PAL was calculated by the following equation. PAL = TEE/BMR [11].

### Assessment of NEAT by using an original questionnaire

After measuring daily PA by the triaxial accelerometer, we asked participants about their daily PA to evaluate NEAT, by using an original questionnaire which we have previously reported (Additional file 1) [7]. The questionnaire consisted of 11 question items about locomotive activities and 25 question items about non-locomotive activities. Table 2 showed questionnaire items about locomotive and non-locomotive activities, and also predicted METs by each activity in our NEAT questionnaire [7,12]. We evaluated each questionnaire item with a score of 1 to 3 points in order of levels of daily PA and then added up the scores to determine the NEAT score.

**Table 2:** Locomotive and non-locomotive activities, and predicted METs by each activity in our NEAT questionnaire

**Locomotive activities**		**Non-locomotive activities**	
**Specific activities**	**Predicted METs**	**Specific activities**	**Predicted METs**
Commuting on foot	4.0	cleaning	2.5-3.8
Walking a lot while working	3.5	cooking	3.5
Getting on a train or a bus	1.3	washing the dishes	3.3
Using stairs	3.5	ironing	1.8
Shopping for food	2.3	cleaning the bath	2.3
Taking the garbage out	2.5	cleaning the garden	4.0
Playing with children outside	3.5-5.8	weeding or gardening	3.5
Going for a walk	3.5	watering plants	1.5
		feeding pets	2.5
		looking after children	2.0-3.0
		looking after the elderly	2.3-4.0
		sewing	1.3
		playing an instrument	2.0

NEAT, non-exercise activity thermogenesis; METs, metabolic equivalent values.

### Statistical analysis

Statistical analysis was performed using SPSS version 19 (IBM Co., Ltd, Chicago, USA). All values were expressed as the mean ± standard deviation (SD). Pearson’s correlation coefficient was calculated in order to analyze the association of the NEAT score with PAL. P value < 0.05 was considered to be statistically significant.

## Results

**Figure 1:**
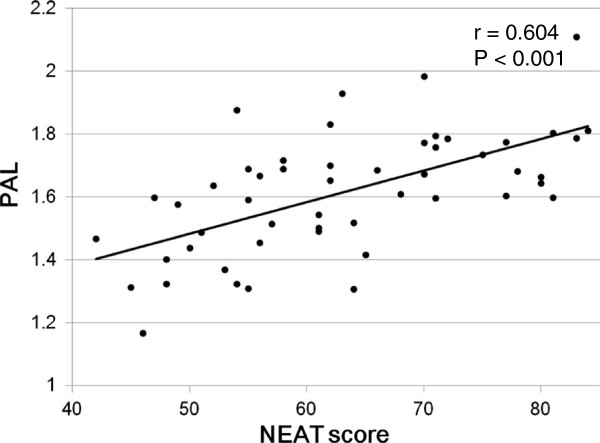
Correlation between the NEAT score and PAL in study participants.

**Figure 2:**
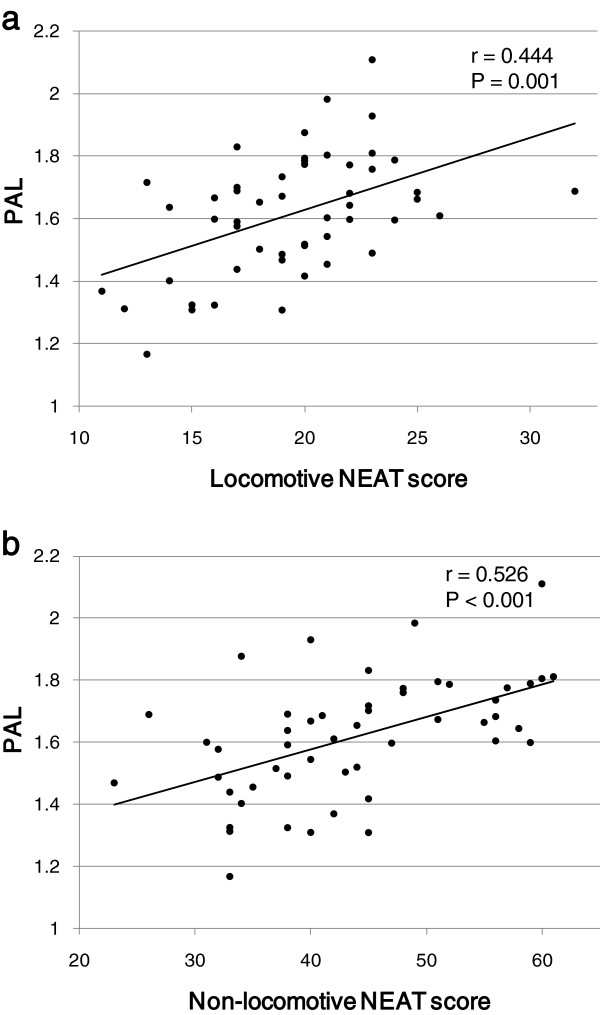
Correlations between PAL and (a) the locomotive NEAT score and (b) the non-locomotive NEAT score.

**Figure 3:**
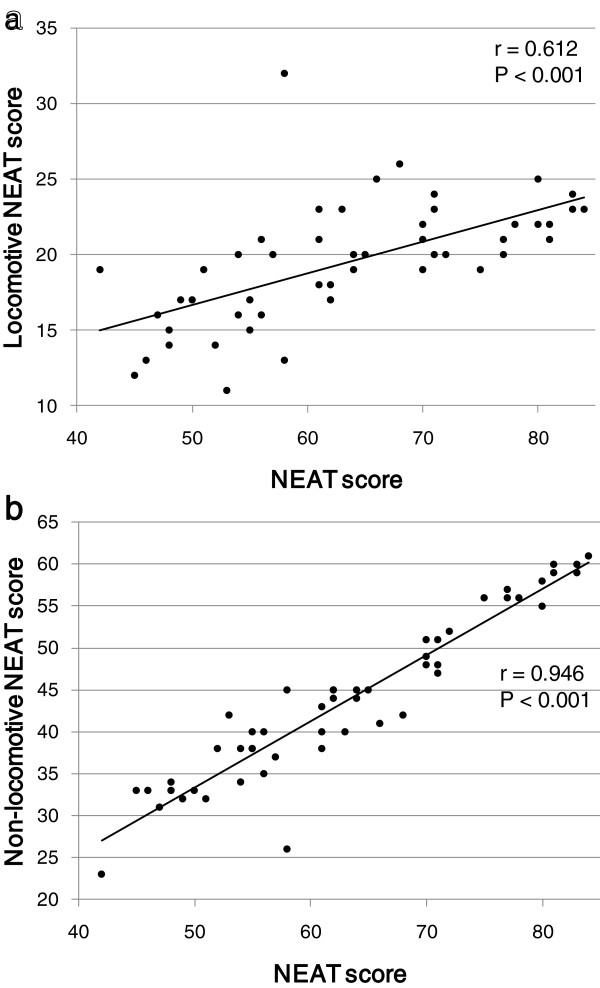
Correlations between the whole NEAT score and (a) the locomotive NEAT score and (b) the non-locomotive NEAT score.

In the present study, the NEAT score was significantly and positively correlated with PAL measured by the triaxial accelerometer (r = 0.604, P < 0.001; Figure 1). PAL was also significantly and positively correlated with both the locomotive NEAT score and the non-locomotive NEAT score (r = 0.444, P = 0.001 and r = 0.526, P < 0.001, respectively; Figure 2). Both the locomotive NEAT score and the non-locomotive NEAT score were significantly correlated with the whole NEAT scores (r = 0.612, P < 0.001 and r = 0.946, P < 0.001, respectively; Figure 3).

Our previous study showed that the NEAT score was significantly and inversely associated with serum insulin levels, suggesting a beneficial association of NEAT with insulin sensitivity in all participants [7] (Table 3). The NEAT score was inversely associated with waist circumference, and also was positively associated with HDL-C level in women [7] (Table 3). However, a beneficial association of NEAT with waist circumference and HDL-C was not observed in men.

**Table 3:** Correlations of NEAT score with physiological and biochemical parameters in all participants, men and women

	**All participants (n = 45)**		**Men (n = 23)**		**Women (n = 22)**	
	**r**	**P value**	**r**	**P value**	**r**	**P value**
Waist circumference	-0.013	0.944	0.460	0.055	-0.509	0.044
Systolic blood pressure	0.184	0.226	0.371	0.082	-0.034	0.882
Diastolic blood pressure	-0.029	0.852	0.108	0.625	-0.137	0.543
Triglycerides	-0.171	0.268	-0.028	0.903	-0.320	0.147
HDL cholesterol	0.079	0.608	-0.441	0.040	0.494	0.020
Serum insulin	-0.420	0.019	-0.422	0.104	-0.421	0.118

r indicates correlation coefficient. HDL, high-density lipoprotein; NEAT, non-exercise activity thermogenesis.

There were no differences in PAL and locomotive NEAT score between men (1.59 ± 0.15 and 20.4 ± 3.9, respectively) and women (1.64 ± 0.22 and 18.6 ± 3.9, respectively). However, the whole NEAT score and non-locomotive NEAT score were significantly higher in women (66.9 ± 12.7 and 48.3 ± 9.2) compared with those in men (59.0 ± 8.6 and 38.6 ± 7.4; P = 0.026 and P < 0.001, respectively).

## Discussion

In the present study, we compared daily PA measured by the triaxial accelerometer with our original NEAT score. To our knowledge, our NEAT questionnaire is the first to evaluate NEAT including locomotive and non-locomotive activities in clinical practices. Our findings showed that the NEAT scores measured by using our questionnaire were highly correlated with PAL measured by the triaxial accelerometer in Japanese patients with type 2 diabetes. Although the NEAT score obtained from the questionnaire is practical and cost-effective to evaluate PA, they are subjective data and may not always represent the true NEAT [13,14]. The accelerometry technique have been found to have a significant correlation with EE measured by the indirect calorimetry, therefore, it has been extensively considered to be a validated method for evaluating PA under free living conditions [9,15-18]. In the present study, the validity of our original NEAT questionnaire was confirmed by using the triaxial accelerometer.

Cooper AR et al. have showed that sedentary time was positively associated with metabolic risks [19], whereas the moderate-to-vigorous-intensity physical activity (MVPA) was associated with reduced metabolic risks [20]. Patients with type 2 diabetes generally show low levels of PA and have difficulty maintaining the recommended PAL [21,22]. The promotion of light-intensity daily PA such as NEAT is more practical and realizable as compared with the promotion of MVPA in the management of type 2 diabetes. We have previously demonstrated that the NEAT score determined by our original questionnaire is favorably associated with insulin sensitivity, abdominal obesity, lipid metabolism and blood pressure in patients with type 2 diabetes [7]. Taking account of present results, the promotion of NEAT may be useful for the management of type 2 diabetes.

We had unsolved things about the NEAT questionnaire in our previous study. The NEAT scores measured by questionnaire were more significantly and beneficially correlated with metabolic parameters in women as compared with those in men [7]. Briefly, the NEAT score was inversely associated with waist circumference, and also was positively associated with HDL-C level in women. However, a beneficial association of NEAT with waist circumference and HDL-C was not observed in men. Our NEAT questionnaire consists of 11 question items about locomotive activities such as walking and going up stairs, and 25 question items about non-locomotive activities such as washing dishes, ironing and sewing, and 21 question items are related to housework. Therefore, our questionnaire can evaluate non-locomotive activities adequately, however, may underestimate locomotive activities. The NEAT score of non-locomotive activities was more strongly correlated with the whole NEAT scores as compared with that of locomotive activities, supporting that our NEAT questionnaire is more excellent to evaluate non-locomotive activities such as housework as compared with locomotive activities such as walking. This may cause different effects of NEAT on metabolic parameters between men and women.

We have to mention the limitations of this study. We might overestimate or underestimate PA in the questionnaire. EE was estimated by regression equation assessed by the triaxial accelerometer. Leenders et al. indicated that the predictive equations based on the relationship between acceleration and EE during locomotive movements led to under- and overestimation of TEE [23]. It is possible that EE and PAL measured by the triaxial accelerometer differ from the true amount. For example, the accelerometer was worn on the left side of the waist and the movements of upper extremities could not be measured completely. The movement of upper extremities contributed less to EE than whole body trunk movements [24], however, our NEAT questionnaire included PA of upper extremities such as washing dishes, ironing and sewing, EE by those activities might have been underestimated.

## Conclusion

We confirmed the validity of NEAT questionnaire by investigating the correlation of the NEAT score to PAL measured by the triaxial accelerometer. Our original NEAT questionnaire may be useful for evaluation of daily PAL in clinical practices.

## Competing interests

The authors declare that they have no competing interests.

## Authors’ contributions

All five authors have substantially contributed to conception and design, acquisition of data or analysis and interpretation of data; drafting the article or revising it critically for important intellectual content; and all authors read and approved the final manuscript.

## Pre-publication history

The pre-publication history for this paper can be accessed here:

http://www.biomedcentral.com/2052-1847/6/27/prepub

## Supplementary Material

Additional file 1 Non-Exercise Activity Thermogenesis (NEAT) score. Click here for file
